# Evaluation of the Efficacy of Supplementation with Planox® Lemon Verbena Extract in Improving Oxidative Stress and Muscle Damage: A Randomized Double-Blind Controlled Trial

**DOI:** 10.7150/ijms.60726

**Published:** 2021-05-03

**Authors:** Mon-Chien Lee, Yi-Ju Hsu, Chin-Shan Ho, Chun-Hao Chang, Ching-Wen Liu, Chi-Chang Huang, Wen-Dee Chiang

**Affiliations:** 1 Graduate Institute of Sports Science, National Taiwan Sport University, Taoyuan City 33301, Taiwan.; 2 Department of Food Science, College of Agriculture, Tunghai University, Taichung City 40704, Taiwan.

**Keywords:** Lemon verbena, oxidative stress, muscle injury, inflammation.

## Abstract

Excessive exercise load can cause muscle soreness and fatigue, as well as inflammation and oxidative stress. Lemon verbena (*Aloysia triphylla; Lippia citriodora*) is often used as a spice in tea or beverages. Its leaves are rich in polyphenols, which have antioxidant and anti-inflammatory bioactivities. In the present study, we investigated whether supplementation with Planox® lemon verbena extract (LVE) could improve muscle damage and biochemical indicators after exhaustive exercise challenge. All subjects (30 males and 30 females) underwent a double-blind trial and were randomly divided into a placebo group (0 mg/human/day) and an LVE supplement group (400 mg/human/day), with gender-equal distribution. All subjects started supplementation 10 days before exhaustive exercise and continued it until all tests were completed. Before the intervention, after the exhaustive exercise, and on the following 3 days, the participants underwent 12-minute Cooper running/walking; blood collection; assessments of pain, muscle stiffness, maximum jump heights, and isometric maximum muscle strength. The results showed that supplementation with LVE effectively increased GPx and reduced CK, IL-6, 8-OHdG and muscle pain after the exhaustive exercise, but it had significant effect on strength recovery. In summary, LVE is a safe and edible natural plant extract that can reduce muscle damage and soreness after exercise. This trial was registered at clinicaltrials.gov as NCT04742244.

## Introduction

Whether one is an untrained or a highly-trained athlete, excessive or unaccustomed exercise can cause the ultrastructural destruction of skeletal muscle fibers, called exercise-induced muscle injury (EIMD) 1. In eccentric contractions especially, when the force exerting pressure on the muscle exceeds the instantaneous force generated by the muscle itself, eccentric (extended) muscle contraction occurs, causing the muscle-tendon system to be forced to extend when contracted 2. Eccentric exercise exerts high mechanical stress on the muscle structure, triggering a series of physiological and morphological changes leading to decreased muscle strength, delayed onset of muscle soreness (DOMS) (usually 12-48 h after exercise and mending within 7 days), muscle swelling and increased concentration of inflammatory biomarkers 3. These effects can cause declines in daily life functions and athletic performance, and even harm to the body.

The DOMS and EIMD caused by exercise can be evaluated from biomechanical indicators, such as reduced maximal isometric contraction or range of motion (ROM) 4 or blood biochemical indicators, such as increased creatine kinase (CK) or lactate dehydrogenase (LDH) 5. In addition, muscle damage may also lead to inflammation and oxidative stress. Inflammatory factors interleukin-6 (IL-6) and Tumor Necrosis Factor-α (TNF-α) 6 or antioxidant indexes such as (superoxide dismutase) SOD and (Glutathione peroxidase) GPx 7 are often used to evaluate adverse reactions in the body. Therefore, the balance of oxidative stress and antioxidant capacity during high-intensity exercise is very important for physiological adaptation. However, according to nutritional supplements and training models, improvements in oxidative stress and inflammation can also improve athletic performance and lead to healthier conditions 8.

Many studies have tried to examine several interventions such as massage 9, cryotherapy 10, cold-water immersion 11, laser acupuncture 12 and nutritional supplement 13, for relieving symptoms or accelerating the recovery of muscle damage or soreness. In recent years, sports nutrition supplements have been very popular, especially those with anti-inflammatory and antioxidant bioactivities, which would reduce fatigue or injury post extreme exercise challenge as well as physical recovery. For examples, polyphenols, resveratrol and curcumin were extracted from fruits, plants and traditional herbal medicine. These food factors have been demonstrated to show effective anti-inflammation on IL-6 expression by regulating nuclear factor-kappa B (NF-kB) 14, 15, 16, or activating the transcription factor Nrf2 to promote the antioxidant effect 17, thereby reducing the effect of eccentric exercise on muscle damage.

Lemon verbena (*A. citrodora*), synonyms: *Aloysia triphylla; Lippia triphylla; Verbena triphylla*, is a perennial subshrub, a species of flowering plant in the verbena family *Verbenaceae*, which is native to South America. It is mainly used in tea, beverages, food, and spices. In the past, it was usually used for gastrointestinal and nervous system diseases in traditional medicine 3. In addition, lemon verbena also has antimicrobial, cardioprotective, neuroprotective, anticonvulsant, anti-inflammatory, and antigenotoxic effects in *in vivo* and *in vitro* tests 18, and be indicated that the supplement of lemon verbena wouldn't cause harm to the human body 19. A variety of phenolic compounds, which are the most relevant biologically active phytochemicals, have been described in lemon verbena aerial part 20. Among these compounds, verbascoside, also known as acteoside, is the most major compound in verbena leaves, and the most effective chemical active ingredient of lemon verbena 21.

Despite many studies have shown that lemon verbena has antioxidant capacity, it can enhance the activity of antioxidant enzymes such as glutathione reductase and glutathione peroxidase, and the ability to diminish protein carbonyls and malondialdehyde 22, only few studies have examined its effects on injury and strength loss caused by exercise 23. Therefore, in the current study, biochemical, biomechanical and physiological tests were used to examine whether supplementation with verbena extract can reduce the damage index and accelerate muscle strength recovery on human.

## Materials and methods

### Subjects

This study was conducted in accordance with the Declaration of Helsinki as a double-blind parallel control trial. Men and women between the ages of 20 and 30 were from the national Taiwan sport university were non-smokers and had no musculoskeletal, medical, metabolic disease, and women not on the reproductive cycle were considered eligible to participate. Exclusion criteria included high blood pressure, asthma, or skeletal neuromuscular injuries in the upper or lower extremities. All the subjects were required to maintain their original exercise and eating habits. During the trial period, they were instructed to avoid taking anti-inflammatory drugs, antioxidants or supplements, and to refrain from exercise training from 48 hours before to 96 hours after the exercise test. The study was approved and reviewed by the Landseed International Hospital Institutional Review Board (Taoyuan, Taiwan; LSHIRB No. 20-016-A2). Before the experiment, the researchers explained the experimental process in detail, and the experiment began after the subjects signed the consent form.

### Experimental Design

**Figure 1:**
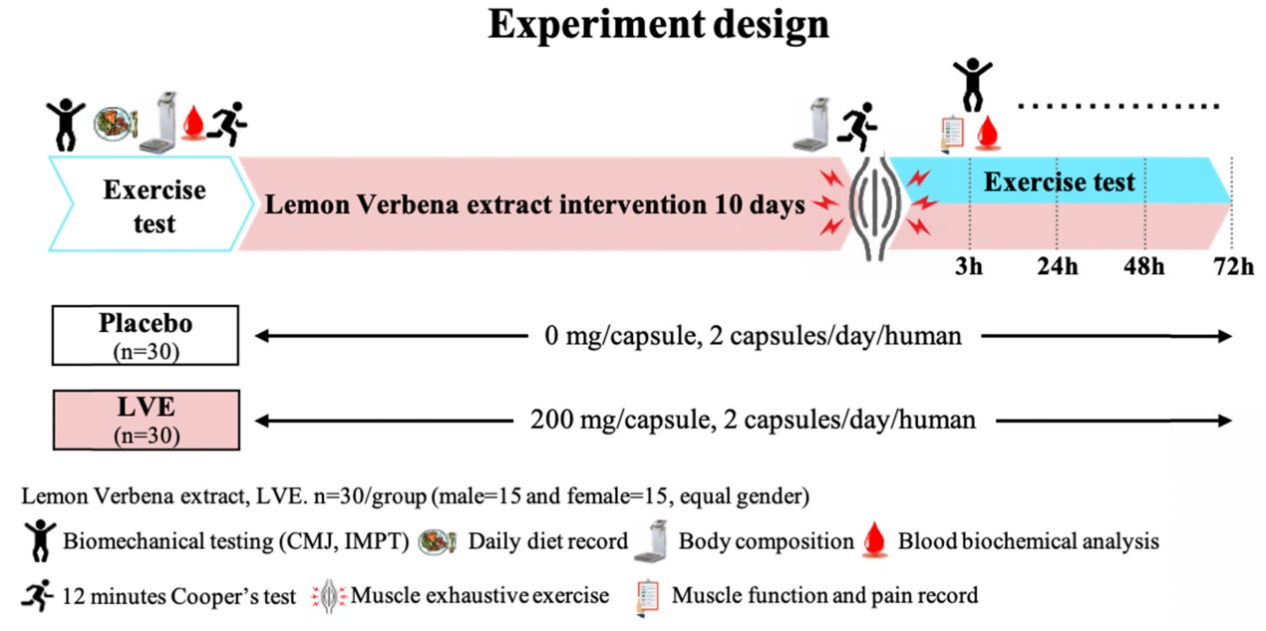
Experimental design. In a randomized, double-blind test, volunteers (30 males and 30 females) were assigned to two groups: a placebo (0 mg/day) group and an LVE (400 mg/day) group. After all subjects received placebo or LVE supplementation for 10 days, they immediately performed exercise endurance challenges and muscle exhaustive exercise. Then at 3, 24, 48, and 72 hours after the muscle exhaustive exercise, all subjects were again subjected to various biomechanical and blood biochemical tests to observe the effects and changes at different time points.

Before the intervention, 30 male and 30 female subjects underwent a double-blind trial and were randomly divided into two groups with gender-equal distribution: a placebo (0 mg/day) group (15 males and 15 females) and an LVE (400 mg/day) group (15 males and 15 females). The experimental process is shown in **Figure 1**. Before the intervention, all subjects performed endurance exercise, biomechanics, basic biochemical values, and body composition tests. After all subjects received placebo or LVE supplementation for 10 days, they immediately performed exercise endurance challenges and muscle exhaustive exercise after be asked fasting 8 hours. Then at 3, 24, 48, and 72 hours after the exhaustive exercise, all subjects were again subjected to various biomechanical and blood biochemical tests to observe the effects and changes at different time points.

### Supplements

**Figure 2:**
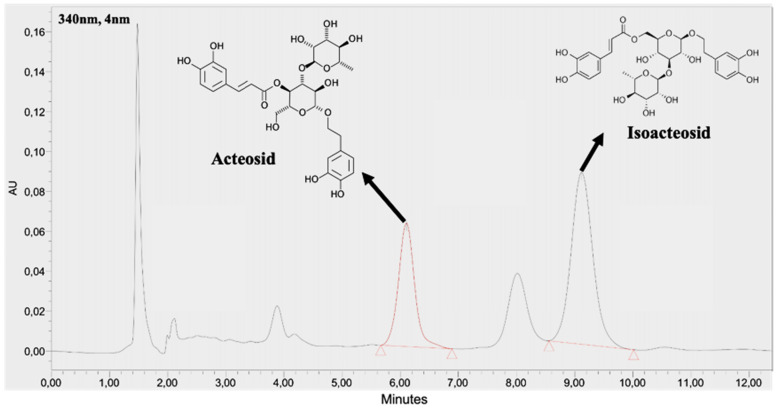
HPLC chromatogram of acteosides and isoactoside in LVE.

The supplement in this study was a commercial product Planox® lemon verbena extract (LVE) (77-1000-00, batch number L019.05.0001), which is obtained by ethanol extraction from organic dried lemon verbena leaves without any additives, and standardized to 9% *acteoside* and *isoactoside*, which were determined by a high-performance liquid chromatography (HPLC) method (BioTeSys GmbH, Esslingen, Germany). As shown on **Figure 2**, The retention time of *acteoside* and *isoactoside* in LVE was 6.10 and 9.12 min, respectively.

Both LVE and placebo were made into capsules of matching size and color, provided by Hänseler AG (Herisau, Switzerland). Each capsule contained 200 mg of lemon verbena extract or placebo (maltodextrin). All subjects were instructed to take two capsules every morning. The product was consumed for the 10 days preceding the exhaustive exercise test, on the day of the test, and for 3 days after the test.

### Muscle Exhaustive Exercise Protocol

The method of muscle damage caused by the maximum eccentric load of the lower extremity was based on past research, with appropriate modifications 24. In the current study, a total of 100 countermovement jumps were performed in 10 sets of 10 jumps every four seconds, with 60 s rest between sets. The knee angle between jumps, 90°, was controlled by the observer. At the end of the test, the Borg rating of perceived exertion (RPE) scale was used to obtain the perceived fatigue level.

### The 12-minute Cooper Running/Walking Test

The 12-minute Cooper running/walking test is used as an initial simple method to assess aerobic endurance and physical fitness 25. The time is recorded from the start of the run, and the distance traveled is recorded every 3 minutes (3rd, 6, 9th and 12th minutes), as previous described 26.

### Countermovement Jump Assessment

The CMJ test is a practical, effective, reliable, and simple method of measuring lower limb strength, which is related to the maximum speed, strength and explosive force of the lower limbs. For this test, participants stood on the Kistler force measurement platform (9260AA, Kistler Co., Ltd., Switzerland) on both feet and performed to inspection. During the test, they were asked to put their hands on their hips and remain on the platform. After that, they were asked to squat down until the knees bent 90 degrees and then to immediately jump as high as possible. The average power (MF), peak power (Fpeak), flight time (FT) and strength development speed (30 ms) were recorded during the jump. Each participant repeated the test 3 times, and CMJ data were obtained at the designated points. The instrument was calibrated for each individual's weight 27. In this study, this assessment was performed before and 24 and 48h after the exhaustive exercise program.

### Isometric Mid-Thigh Pull (IMTP)

The IMTP is a force-time diagnostic tool 28. Customized IMTP test equipment and two force plates (type 9287BA, Kistler Instruments AG, Winterthur, Switzerland) were used. All participants stood with their feet the same width apart, and the rod was placed between the thighs, with the torso upright, the spine neutral, and the knee and hip angles at 140°, to familiarize the participants with the IMPT test method. We confirmed that the force trace of the practice test was remains symmetrical and the weighing cycle was stable. Each test phase repeated each participant's specific measurement at 2-minute intervals to reduce the risk of measurement errors related to changes in posture. The average absolute peak force (PF), relative peak force (N/Kg), rate of force development (RFD), peak rate of force development (pRFD), time to peak force (TPF) and time to peak rate of force development (TPRFD) parameters were recorded. This assessment was performed before and 24 and 48h after the exhaustive exercise program.

### Muscle Stiffness Test

A handheld MyotonPRO (Myoton Ltd., Tallinn, Estonia) device was applied to measure the viscoelastic and stiffness response of the muscle to a brief (15 ms) mechanical impulse (force, 0.4 N) on the skin surface above the muscle. It is the biomechanical property of a muscle that resists either contraction or an external force, tending to deform its initial shape. The probe of MyotonPRO, placed perpendicular to the skin surface overlying tested muscle, produces a short mechanical impulse. Naturally damped oscillations due to the response of soft tissue are then processed by the device. The inner and outer femoral muscles of the thigh were tested at the midpoint of the muscle. In this study, five measurements were taken and the average value was used. During the MyotonPRO measurement, under the guidance of the researcher, the participants were asked to lie supine on the bed and to relax their muscles 29.

### Assessment of Pain Caused by Exercise (VAS)

For the pain assessment, the subjects were asked to sit on a chair or to stand up from a chair and to assess the pain they experienced during the process on a 100 mm visual analog scale (VAS) ranging from 0 mm (no pain) to 100 mm (worst imaginable pain). This assessment was performed before and 3, 24, 48, and 72 h after the exhaustive exercise program.

### Body Composition

We used the InBody 570 (In-body, Seoul, South Korea), the bioelectrical impedance analyzer (BIA) to measure body composition. The device can screen frequencies of 1, 5, 50, 260, 500 and 1000 kHz within 60 seconds. After cleaning the palms and soles of the feet, the subject stood vertically on the electrodes of the instrument, holding the sensing handle with both hands and keeping the arms away from the body at a 30° angle, and avoided talking or moving during the measurement 26. We would measurement about body weight (kg), BMI (kg/cm2), fat mass (%), and lean mass (kg).

### Serum Biochemical Analysis

To understand the subject's metabolism and health before the intervention, we collected blood from a venous catheter in the arm for analysis at the beginning of the experiment. The physiological adaptations and clinical biochemistry of the blood serum were assessed with an autoanalyzer (Hitachi 7060, Tokyo, Japan) for glucose, aspartate transaminase (AST), aminotransferase (ALT), LDH, total cholesterol (TC), triglyceride (TG), high-density lipid-cholesterol (HDL-C), and low-density lipid-cholesterol (LDL-C) levels.

### Muscle Damage and Antioxidative Capacity Biochemical Analysis

CK is a biomarker of muscle damage and usually increases after strenuous exercise 30. In our study, CK levels were determined with an autoanalyzer (Hitachi 7060, Tokyo, Japan) from blood samples obtained before and 3, 24, and 48 hours after the exhaustive exercise regimen.

Exercise training would generate oxidative stress and regulate the endogenous antioxidant defense system (including GPx) 31. The analysis of plasma collected pre-exercise and 3 and 48 h post exercise was performed with the GPx-Assay-Kit (Cayman Chemical Company, Ann Arbor, Michigan, USA).

IL-6 is a multifunctional cytokine involved in pro-inflammatory and anti-inflammatory processes 32. The IL-6 response caused by exercise depends on the intensity and duration of the exercise. The analysis of serum collected pre-exercise and 3 and 48 h post exercise was performed with the IL-6 Quantikine HS ELISA Kit (R&D Systems, Inc. Minneapolis, MN, USA).

### Determination of 8-Hydroxy-2'-Deoxyguanosine (8-OHdG) in Urine

For the urinary 8-OHdG measurement, urine samples were collected at 24 hours after the muscle exhaustive exercise, and frozen immediately at the survey site and stored at -20°C until analysis. 8-OHdG was measured by enzyme-linked immunosorbent assay (ELISA) using a commercial analysis kit (Cayman Chemical Company, Ann Arbor, Michigan, USA).

### Statistical Analysis

All the data are expressed as mean ± SEM. Statistical analyses were performed in SAS 9.0 (SAS Inst., Cary, NC, USA). Multi-group comparisons were analyzed by one-way analysis of variance (ANOVA), and within-group differences (before vs. after LVE supplementation), by paired Student's t-test. Statistical significance was set at *p* < 0.05.

## Results

### Subjects' Basic Information Data

**Table 1** presents the basic information of the subjects and the biochemical values ​​of basal metabolism. The average ages of the subjects in the placebo and LVE supplement groups were similar, 22.1 and 22.3 years old, respectively. The average heights were also quite close. In terms of metabolism, blood biochemical values ​​including glucose, TC, TG, HDL, LDL and other indicators had no significant differences between the two groups, all falling within the normal healthy range.

### Effect of LVE Supplementation on Distance in 12-minute Cooper Running/walking Test

**Figure 3:**
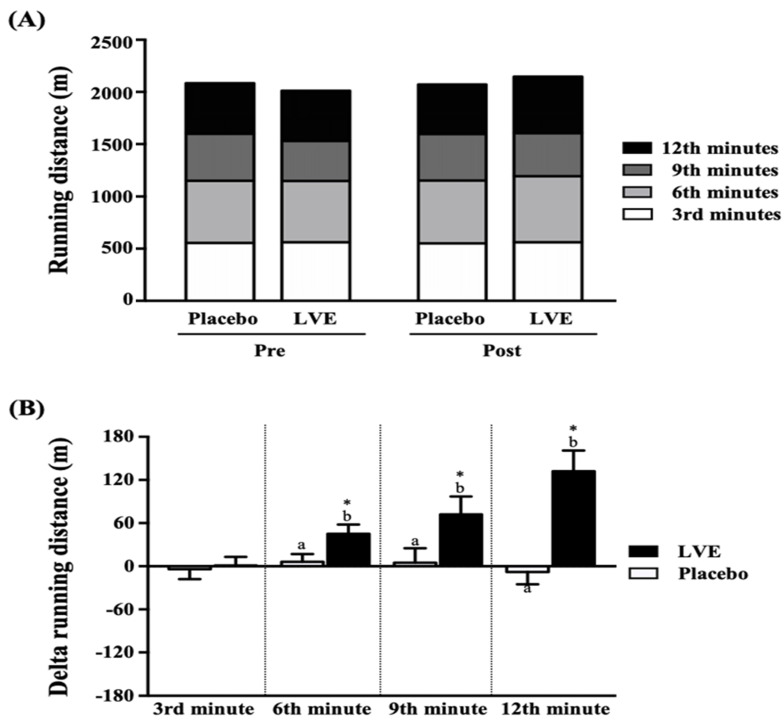
Effects of LVE supplementation for 10 days on (A) running distance and (B) change in running distance. Data are presented as mean ± SEM. Different superscript letters (a, b) indicate significant difference at p < 0.05, and the baseline compared with each time point (3rd, 6th, 9th, and 12th min). Administration effects were statistically analyzed with a paired Student's t-test, * *p* < 0.05.

As shown in **Figure 3A**, the placebo group and the LVE supplement group were tested for endurance with a 12-minute Cooper running/walking test pre- and post-intervention, and there was no significant difference in running distance between the two groups. However, after comparing the changes in the values ​​of the two groups between pre- and post-intervention (**Figure 3B**), we found that the running distance changes in the placebo group and the LVE supplement group at the 3rd, 6th, 9th, and 12th min were -4 ± 14 and 1 ± 12 m, 6 ± 11 and 45 ± 13 m, 5 ± 20 and 72 ± 25 m, and -8 ± 17 and 132 ± 29 m, respectively. Compared with those in the placebo group, the improvements in the LVE group were significantly higher at the 6^th^ min (*p* = 0.0257), 9^th^ min (*p* = 0.0393) and 12^th^ min (*p* = 0.0001). In the within-subject pre- and post-intervention comparison, only in the LVE supplement group was there significant improvement at the 6^th^ (*p* = 0.0257), 9^th^ (*p* = 0.0257) and 12^th^ (*p* = 0.0257) minutes.

### Effect of LVE Supplementation on the Assessment of Muscle Soreness

The effect of exhaustive exercise on muscle soreness was measured with objective instruments for determining muscle tension, stiffness and elasticity, and recorded with a subjective perception scale by VAS. We analyzed two important muscle groups, the medial and lateral quadriceps. **Table 2** shows that muscle tension in the medial quadriceps muscle was less affected by exhaustive exercise but that it increased with time in the lateral quadriceps muscle. In both the medial and lateral quadriceps at 48 ​​hours after the exhaustive exercise, muscle tension was significantly higher in the placebo group than in the LVE group (*p* < 0.05). Only 3 hours after the exhaustive exercise, the muscle stiffness in the medial and lateral quadriceps was significantly greater in the placebo group than in the LVE group. However, supplementing with LVE had no significant effect on muscle elasticity.

In the VAS evaluation (**Table 3**), the muscle pain appearing within 48 and 72 hours after the excessive exercise of the medial quadriceps muscle was significantly lower in the LVE group than in the placebo group. In the lateral quadriceps muscle, muscle pain was significantly lower in the LVE group than in the placebo group at 24, 48, and 72 hours after the exhaustive exercise.

### Effect of LVE Supplementation on Muscle Strength and Jumping Force

The CMJ test, force plate and high-speed camera were used to understand the effects of LVE supplementation on muscle strength and jumping ability after the exhaustive exercise. As shown on the** Table 4**, jumping force decreased 24 hours after the exhaustive exercise and gradually recovered after 48 hours. However, there were no significant differences between the placebo and LVE groups.

### Effect of LVE Supplementation on the Isometric Maximum Muscle Strength

After the exhaustive exercise, the isometric maximum muscle strength recovery effect was better in the LVE group than in the placebo group, but still less pronounced (**Table 5**).

### Effect of LVE Supplementation on Creatine Kinase

**Figure 4:**
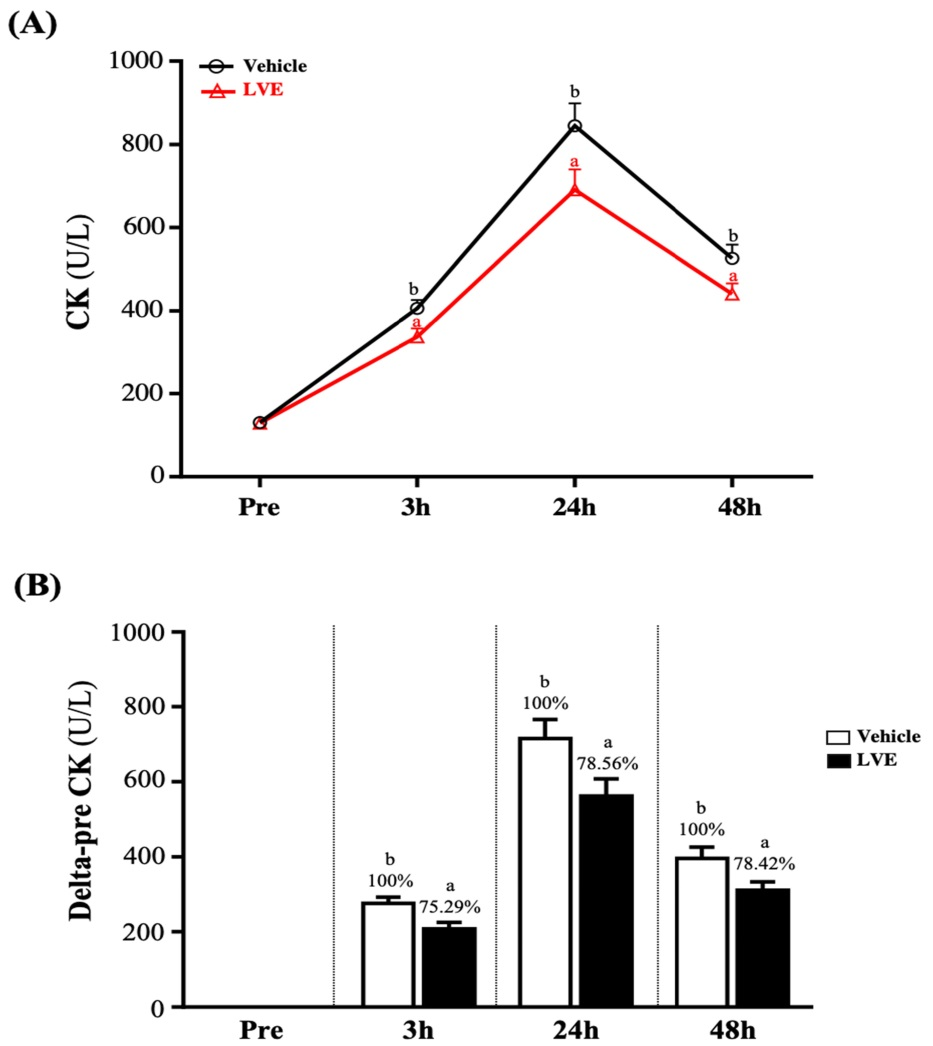
Effects of LVE supplementation for 10 days on (A) CK activity and (B) delta to pre-test. Data are presented as mean ± SEM. Different superscript letters (a, b) indicate significant difference at *p* < 0.05. CK, creatine kinase.

The change in the CK value of muscle injury induced by exhaustive exercise is shown in **Figure 4A**. There was no significant difference between the two groups before exhaustive exercise. As time increased, the activity of CK gradually increased until it peaked 24 hours after the exhaustive exercise, after which it began to decline and recover. However, at 3, 24, and 48 hours after the exhaustive exercise, CK was significantly higher in the placebo group than in the LVE group.** Figure 4B** shows the results for the placebo group and the LVE group at 3, 24, and 48 hours after the exhaustive exercise. Compared with the pre-test, significant differences between the two groups were noted. At 3, 24, and 48 hours after the exhaustive exercise, in the LVE group, the activity of CK decreased significantly, by 24.71% (*p* = 0.0073), 21.44% (*p* = 0.0288) and 21.58% (*p* = 0.0271), respectively.

### Effect of LVE Supplementation on Inflammation and Antioxidant Levels

**Figure 5:**
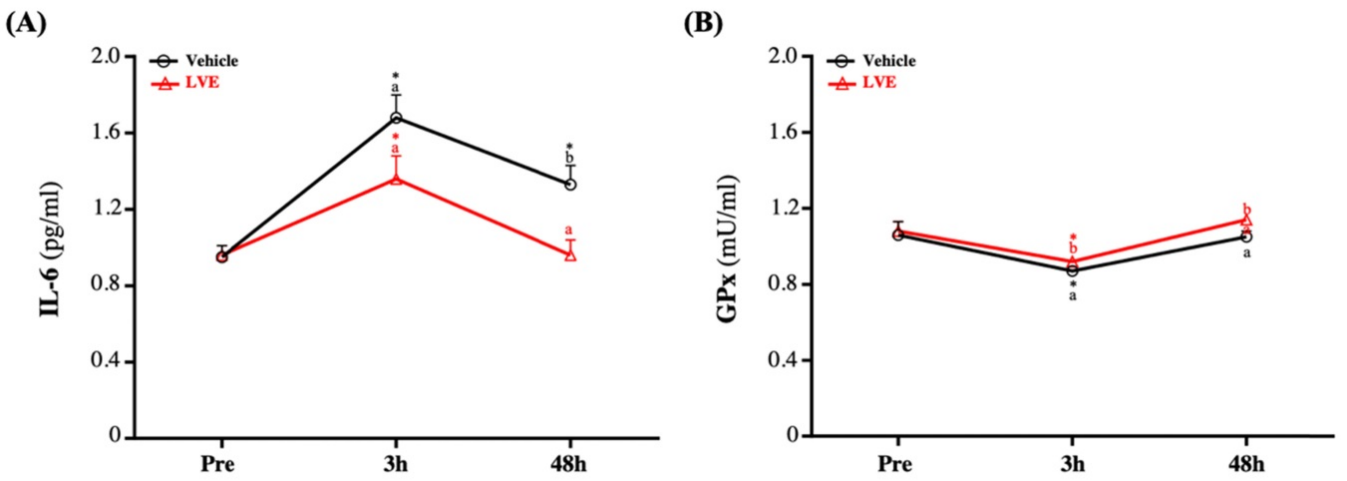
Effects of LVE supplementation for 10 days on (A) IL-6 and (B) GPx. Data are presented as mean ± SEM. Different superscript letters (a, b) indicate significant difference at *p* < 0.05, and baseline is compared with post-3h, 48h, respectively. Administration effects were statistically analyzed with a paired Student's t-test, * p < 0.05. IL-6, interleukin-6; GPx, glutathione peroxidase.

Before the exhaustive exercise, there was no significant difference in serum IL-6 between the placebo group and the LVE group. At 48 hours after the exercise, the serum IL-6 level was significantly higher in the placebo group than in the LVE group (*p* = 0.0053). In addition, in terms of within-subject comparisons, the serum IL-6 level of the placebo group significantly increased at 3 (*p* < 0.0001) and 48 (*p* = 0.0021) hours after the exercise relative to the baseline (pre-exercise). In contrast, the serum IL-6 level of the LVE group was significantly higher than the baseline only at 3 hours after the exercise (*p* = 0.0002) (**Figure 5A**).

Although there was no significant difference in GPx between groups before the exercise, it decreased slightly and then recovered after the exercise. However, GPx was still significantly higher in the supplementary LVE group than in the placebo group at 3 (*p* = 0.0446) and 48 (*p* = 0.0300) hours after the exercise. In addition, in the within-group comparisons, the placebo (*p* = 0.0167) and the LVE (*p* = 0.0047) groups only had significant differences before and 3 hours after the exercise (**Figure 5B**).

### Effect of LVE Supplementation on 8-Hydroxy-2'-Deoxyguanosine (8-OHdG) in Urine

**Figure 6:**
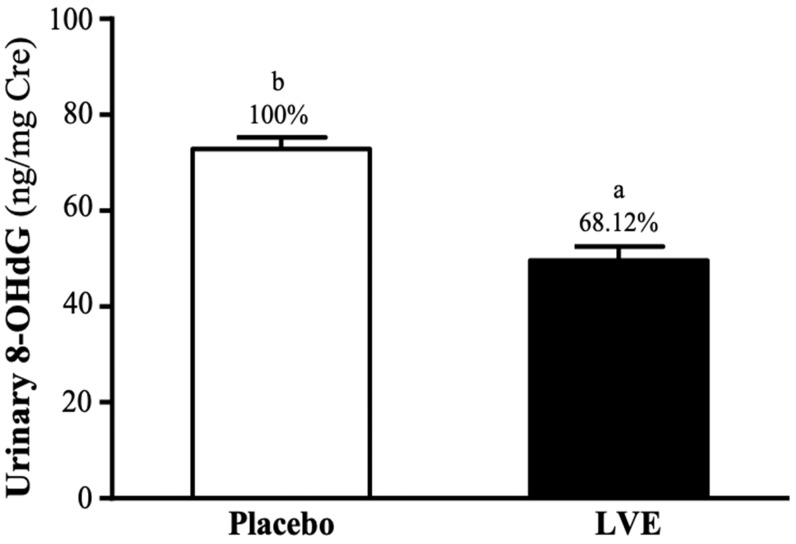
Effects of LVE supplementation for 10 days on urinary 8-OHdG. Data are presented as mean ± SEM. Different superscript letters (a, b) indicate significant difference at *p* < 0.05. 8-OHdG, 8-hydroxy-2'-deoxyguanosine.

8-OHdG in urine is often used as an indicator of deoxyribonucleic acid (DNA) damage caused by oxidative stress. In this study, subjects' urine was collected 24 hours after the exhaustive exercise for analysis. As shown on **Figure 6**, the 8-OHdG levels in the placebo and LVE groups were 72.85 ± 2.45 and 49.62 ± 2.92 (ng/mg Cre), respectively. The level was significantly lower in the LVE group (31.88%; *p* = 0.0001) than in the placebo group.

### Effect of LVE Supplementation on the blood biochemical

In the blood biochemical, serum glucose, AST, ALT and LDH in the different time point were no significantly between placebo and LVE group, respectively (**Table 6**).

## Discussion

LVE is a complex extraction formula, containing polyphenol-like substances and many more not yet characterized components. Therefore, possibly, different mechanism was involved in the muscle-protecting effects. This study explored the effects of a lemon verbena extract supplement containing 9%* acteoside* and isoacteoside in reducing muscle damage and promoting muscle strength recovery. Based on the results, we found that although the supplementation of lemon verbena extract produced no significant difference in strength performance, it effectively reduced muscle damage indicators and indicating possible pro-inflammatory stress caused by exercise, such as CK and IL-6 33. In addition, subjects' subjective feelings and objective detection of pain were also significantly improved.

Many previous studies have used polyphenols as acute and non-acute sports nutrition supplements. They have antioxidant and anti-inflammatory effects, and they eliminate reactive oxygen species (ROS) produced after exercise, which may be one of the factors that improve exercise performance [34)]. In addition, both acute and chronic supplementation of polyphenols can improve vascular function, especially endothelium-dependent vasodilation. Endothelial-dependent vasodilation is nitric oxide (NO)-dependent. The main mechanism of NO is to inhibit the vasoconstriction of adrenaline through direct vasodilation of vascular smooth muscle cells and increased muscle implantation 35. Therefore, it is possible to increase aerobic metabolism by increasing oxygen supply to muscle cells and higher nutrient supply and removal of metabolites 36. NO may also affect muscle glucose metabolism, especially by increasing muscle absorption. The endogenous NO produced by calcium^2+^ in the sarcoplasmic reticulum may improve muscle contractility 37. Past studies have confirmed that supplementing with polyphenol-containing nutritional supplements can effectively increase maximum oxygen uptake, exercise performance, and exhaustion time 34. In the current study, to avoid placing too much burden on the subjects and not affect the other experiments, we used the 12-minute Cooper running/walking test to understand the effect of supplementing with LVE containing polyphenol compounds on exercise endurance performance. The only component of the placebo was maltodextrin, which is a legal food additive and is often used as a placebo in experiments. Previous studies focused on the use of maltodextrin as a carbohydrate supplement as an energy source and found that it does not affect sports performance 38. The results showed that although there was no significant difference in endurance time between the placebo group and the LVE group, the progress of the latter was significantly better in terms of running distance at each time point (**Figure 3**).

CK is a dimeric protein with an enzyme that can catalyze the transfer of high-energy phosphate from ATP to creatine, and it is important in maintaining cell energy and muscle cell metabolism. It mainly exists in the heart muscle and is rare in other tissues, such as skeletal muscle. CK is the main isoenzyme in skeletal muscle; it binds to the M-line structure of muscle tissue in myofibrils. In normal serum, the total CK level mainly comes from skeletal muscle 39. During muscle contraction, adenosine triphosphate (ATP) is consumed to form adenosine diphosphate (ADP), and the enzyme is phosphorylated to ATP again by the CK enzyme (creatine phosphate is used as a phosphate donor) 40. Cell membrane rupture caused by hypoxia or other damage releases CK from the cytoplasm into the systemic circulation. In tissue damage involving skeletal and cardiac muscle, and in brain damage, serum CK activity increases 41. The concentration of CK starts to increase within 2-12 hours after the onset of muscle damage, reaches a maximum value in 24-72 hours, and decreases as the muscle damage subsides within 2-5 days 39. Therefore, the appearance of CK in the blood is generally considered an indirect sign of muscle damage. The other indicator of muscle damage is IL-6. Many aspects of IL-6-mediated exercise-induced acute phase responses include the upregulation of the antioxidant defense system in response to oxidative stress. After intense physical exercise, IL-6 is synthesized by actively contracting muscle fibers. The maximum IL-6 level is found immediately after the exercise, and then it drops rapidly 33. Although many studies clearly show that supplementation with polyphenols can effectively reduce injury indicators caused by exercise, such as CK and IL-6, this seems to be related to reducing oxidative stress, and antioxidant and anti-inflammatory effects. However, it is unclear whether this mechanism has been confirmed 42. Nevertheless, compared with the placebo group, supplementation with LVE containing polyphenols in this study significantly reduced the peak injury indexes of CK (**Figure 4**) and IL-6 (**Figure 5A**) after the exercise and led to better recovery.

It is well known that acute exhaustive exercise will increase the production of ROS, blood oxidative stress and total oxidant biomarkers, and that this production increases with greater exercise time and exercise intensity 43. According to reports, low concentrations (i.e., resting) of human glutathione (GSH) levels were related to decreased physical fitness, increased oxidative stress and impaired red blood cell redox metabolism 44. Among them, the most reasonable vascular mechanism is to reduce the production of ROS or increase the detoxification ability of ROS through the antioxidant system 34. Since the reaction between peroxide and NO will reduce the production of peroxynitrite, reduced ROS exposure will improve the bioavailability of the effective vasodilator NO. In addition, due to the activation of muscle tissue damage, inflammatory cells (such as neutrophils and lymphocytes) during exercise will further increase overgrowth, which may lead to direct damage to DNA. Although O2·- and NO are the main free radicals produced by skeletal muscle contraction, they do not directly damage DNA 44. This is the reaction of OH with different components of DNA (such as DNA bases and deoxyribose), which are destroyed by hydrogenation or extraction of hydrogen, resulting in various products; single-strand and/or double-strand breaks; tandem damage; and DNA-protein interaction, which have combined effects 45, 46. Among the four DNA bases, guanine has the smallest reduction potential, is an excellent electron donor, and is most susceptible to OH oxidation 46. DNA damage is usually repaired by base excision and repair, while oxidation products are excreted from the body through urine. 8-OHdG is the most common DNA damage biomarker in urine and blood samples, and one of the most widely studied oxidative metabolites, and is considered a biomarker of DNA oxidative damage 47. In the current study, 8-OHdG in urine were selected as parameters that provide information on oxidative damage. Our results showed that the urine 8-OHdG index (**Figure 6**) had lower oxidative damage.

Previously study had pointed out that strenuous exercise could increase free radicals in skeletal muscles, caused GSH oxidation, and released lysozymes and other signals that damage cells 48. GSH redox cycle is a major source of protection against mild oxidative stress, whereas catalase (CAT) becomes increasingly important in protection against severe oxidative stress 49. However, in animal cells, and especially in human erythrocytes, the principal antioxidant enzyme for H_2_O_2_ detoxification has for a long time been considered to be GPx 50. GPx is a general term for a family of enzymes with peroxidase activity, and its main biological function is to protect organisms from oxidative damage. Mainly activate glutathione, directly reduce lipid peroxide R-OOH to alcohol R-OH or fraction decomposition H_2_O_2_, GPX competed with catalase for H_2_O_2_ as a substrate, but catalase had much lower affinity for H_2_O_2_ than GPX 51. Therefore, in the current study, glutathione peroxidase in plasma was selected as a parameter to provide information on antioxidant capacity. As shown on **Figure 5B**, although GPx activity decreased after the exercise and then recovered, compared with the placebo group, the LVE supplement group had better antioxidant capacity.

Muscle soreness peaks 24 hours or 48 hours after injury exercise 3. In the current study, muscle damage caused by exhaustive exercise had a subjective feeling to the subjects. Muscle stiffness and elasticity seemed to have trends resembling those of the blood damage indicators, but there did not appear to be a significant effect on muscle strength. Before and after the exhaustive exercise, we conducted explosive strength, isotonic muscle strength, anaerobic capacity and fatigue index tests. All values ​​began to decline 24 hours after the exercise and gradually recovered after 48 hours. However, there was no significant difference between the placebo group and the LVE group. As in previous retrospective studies, supplementation of nutritional supplements with antioxidant effects had no direct effect on strength recovery after muscle injury. Insufficient intensity of exhaustive exercise or the subject's pain tolerance and habits may limit exercise 52.

In this study, one limitation was that it was necessary to guarantee subjects returned to the laboratory for various tests at 3, 24, 48, and 72 hours after the exhaustive exercise. Therefore, out of 98 subjects who met the criteria, only 69 who agreed to participate on time were recruited. Of these, 9 subjects withdrew due to temporary conditions, and in the end, 60 subjects completed the study. Whether physiological factors have impacts on the outcomes examined in this experiment needs to be further determined.

## Conclusions

In conclusion, the current study supplementation with lemon verbena extract containing polyphenols for 10 days effectively reduced muscle damage and oxidative stress after exhaustive exercise. The benefits on strength recovery need to be further explored. In addition, LVE increased antioxidant activity and reduced pain and muscle stiffness.

## Figures and Tables

**Table 1:** Basic information data of the subjects.

Characteristics	Placebo	LVE
**Age** (y)	21.0 ± 0.4	21.3 ± 0.4
**Height** (cm)	167.5 ± 1.1	167.8 ± 1.7
**Weight** (kg)	62.7 ± 1.3	62.7 ± 1.9
**BMI** (kg/m^2^)	22.3 ± 0.4	22.1 ± 0.4
**LBM** (kg)	27.9 ± 1.0	28.0 ± 1.2
**FBM (%)**	20.6 ± 1.3	20.6 ± 1.4
**Glucose** (mg/dL)	87.0 ± 1.0	87.0 ± 1.0
**TC** (mg/dL)	175.0 ± 4.0	175.0 ± 4.0
**TG** (mg/dL)	67.0 ± 3.0	67.0 ± 2.0
**HDL** (mg/dL)	66.1 ± 1.4	66.2 ± 1.4
**LDL** (mg/dL)	86.5 ± 2.5	86.6 ± 3.4

Data are expressed as mean ± SEM. There were no significant group differences in the basic information data. BMI, body mass index; LBM, lean body mass; FBM, fat body mass; TC, total cholesterol; TG, triglyceride; HDL, high-density lipid; LDL, low-density lipid.

**Table 2:** The LVE supplementation on the assessment of muscle soreness by Myoton.

Characteristics	Frequency [Hz]		Stiffness [N/m]		Decrement	
	Placebo	LVE	Placebo	LVE	Placebo	LVE
**Medial quadriceps**						
**Baseline**	13.5 ± 0.2	13.5 ±0.2	204 ± 5	204 ± 6	1.10 ± 0.0	1.10 ± 0.0
**3h**	13.4 ± 0.1	13.2 ± 0.2	201 ± 5^b^	193 ± 6^a^	1.12 ± 0.0	1.10 ± 0.0
**24h**	13.7 ± 0.1	13.2 ± 0.2	211 ± 5	196 ± 6	1.11 ± 0.0	1.05 ± 0.0
**48h**	13.5 ± 0.2^b^	13.3 ± 0.2^a^	186 ± 6^*^	175 ± 5^*^	1.08 ± 0.0	1.04 ± 0.0
**Lateral quadriceps**						
**Baseline**	15.8 ± 0.2	15.7 ± 0.4	280 ± 6	280 ± 9	1.37 ± 0.0	1.36 ± 0.1
**3h**	16.4 ± 0.3^*^	16.0 ± 0.3	291 ± 7^b^	274 ± 5^a^	1.51 ± 0.0	1.37 ± 0.0
**24h**	17.7 ± 0.4^*^	16.7 ± 0.3	303 ± 9^*^	306 ± 8	1.45 ± 0.1	1.39 ± 0.0
**48h**	17.9 ± 0.4^b,*^	16.5 ± 0.2^a^	303 ± 8^*^	285 ± 7	1.40 ± 0.0	1.31 ± 0.0

Data are presented as mean ± SEM. Different superscript letters (a, b) indicate significant difference between groups at *p* < 0.05, and baseline is compared with post-3h, 24h, 48h, respectively. Administration effects were statistically analyzed with a paired Student's *t*-test, compared with baseline, * *p* < 0.05.

**Table 3:** The VAS score to assess muscle pain.

Characteristics	Medial quadriceps		Lateral quadriceps	
	Placebo	LVE	Placebo	LVE
**Baseline**	0.0 ± 0.0	0.0 ± 0.0	0.0 ± 0.0	0.0 ± 0.0
**3h**	2.3 ± 0.4^*^	1.9 ± 0.4^*^	2.3 ± 0.4^*^	1.9 ± 0.4*
**24h**	4.0 ± 0.4^*^	3.3 ± 0.4^*^	4.1 ± 0.4^b,*^	2.7 ± 0.4^a,*^
**48h**	4.4 ± 0.4^b,*^	2.5 ± 0.4^a,*^	4.0 ± 0.4^b,*^	2.0 ± 0.3^a,*^
**72h**	3.0 ± 0.4^*^	1.5 ± 0.3^*^	2.8 ± 0.4^b,*^	1.3 ± 0.3^a,*^

Data are presented as mean ± SEM. Different superscript letters (a, b) indicate significant difference between groups at *p* < 0.05, and baseline is compared with post-3h, 24h, 48h, and 72h, respectively. Administration effects were statistically analyzed with a paired Student's t-test, compared with baseline, * *p* < 0.05.

**Table 4:** LVE supplementation effects on muscle strength and jumping force.

Characteristics	Baseline		24h		48h	
	Placebo	LVE	Placebo	LVE	Placebo	LVE
**RFD 30ms max** (N/Kg*Sec)	11.08 ± 0.50	11.17 ± 0.58	9.14 ± 0.53^a,*^	10.69 ± 0.56^b^	10.39 ± 0.66	10.91 ± 0.71
**RFD** (N/Kg*Sec)	3.67 ± 0.31	3.68 ± 0.29	3.20 ± 0.25^*^	3.21 ± 0.21	3.32 ± 0.29^*^	3.46 ± 0.25
**Time to force peak** (Sec)	0.52 ± 0.04	0.52 ± 0.04	0.63 ± 0.03^*^	0.60 ± 0.03^*^	0.58 ± 0.03	0.60 ± 0.03^*^
**Force peak** (N)	833 ± 38	838 ± 45	745 ± 29^*^	792 ± 42^*^	756 ± 28^*^	796 ± 43^*^
**Relative net impulse** (N*Sec/Kg)	9.55 ± 0.23	9.52 ± 0.26	10.32 ± 0.21^*^	10.14 ± 0.22^*^	10.02 ± 0.20^*^	9.88 ± 0.23
**Flight time** (Sec)	0.52 ± 0.01	0.53 ± 0.02	0.53 ± 0.01	0.53 ± 0.02	0.53 ± 0.01	0.52 ± 0.01
**Jump height** (mm)	508 ± 23	507 ± 24	504 ± 19	512 ± 23	501 ± 19	509 ± 22

Data are presented as mean ± SEM. Different superscript letters (a, b) indicate significant difference between groups at *p* < 0.05, and baseline is compared with post-, 24h, and 48h, respectively. Administration effects were statistically analyzed with a paired Student's t-test, compared with baseline, * *p* < 0.05. RFD, rate of force development.

**Table 5:** LVE supplementation effect on isometric maximum muscle strength.

Characteristics	Baseline		24h		48h	
	Placebo	LVE	Placebo	LVE	Placebo	LVE
**Peak force** (N)	1190 ± 78	1198 ± 84	1024 ± 62^*^	1135 ± 73	1059 ± 58^*^	1172 ± 76
**Relative peak power** (N/Kg)	17.98 ± 1.12	18.12 ± 1.11	14.80 ± 0.87^*^	15.54 ± 0.82^*^	16.49 ± 0.99^*^	17.80 ± 0.99
**pRFD** (N/S)	7145 ± 794	7127 ± 590	6486 ± 595	6737 ± 504	6999 ± 530	7677 ± 682
**Time to peak force** (S)	2.48 ± 0.14	2.50 ± 0.16	3.24 ± 0.18^*^	2.71 ± 0.19	2.98 ± 0.23	2.46 ± 0.14
**Time to peak RFD** (S)	0.17 ± 0.01	0.17 ± 0.01	0.2 ± 0.05	0.19 ± 0.03	0.20 ± 0.03	0.17 ± 0.01

Data are presented as mean ± SEM. Different superscript letters (a, b) indicate significant difference between groups at *p* < 0.05, and baseline is compared with post-, 24h, and 48h, respectively. Administration effects were statistically analyzed with a paired Student's t-test, compared with baseline, * *p* < 0.05. RFD, rate of force development.

**Table 6:** LVE supplementation effect on the blood biochemical.

Characteristics	Baseline		3h		24h		48h	
	Placebo	LVE	Placebo	LVE	Placebo	LVE	Placebo	LVE
**Glucose** (md/dL)	87 ± 1	87 ± 1	95 ± 1	95 ± 2	101 ± 1	101 ± 1	99 ± 1	98 ± 1
**AST** (U/L)	27 ± 1	27 ± 1	27 ± 1	27 ± 2	26 ± 1	27 ± 2	28 ± 1	28 ± 2
**ALT** (U/L)	21 ± 1	22 ± 2	21 ± 1	20 ± 1	19 ± 1	17 ± 1	17 ± 1	20 ± 2
**LDH** (U/L)	460 ± 8	442 ± 11	445 ± 6	441 ± 9	437 ± 6	420 ± 9	438 ± 7	428 ± 12

Data are presented as mean ± SEM. AST, aspartate transaminase; ALT, alanine transaminase; LDH, lactate dehydrogenase.
